# Health literacy among fathers and fathers-to-be: a multi-country, cross-sectional survey

**DOI:** 10.1093/heapro/daad131

**Published:** 2023-10-18

**Authors:** Karen Wynter, Vanessa Watkins, Shane Kavanagh, Sarah Hosking, Bodil Rasmussen, Helle Terkildsen Maindal, Jacqui Macdonald

**Affiliations:** Department of Psychiatry, School of Clinical Sciences, Monash University, Level 3, P Block, 246 Clayton Road, Clatyon, Victoria, 3168, Australia; School of Nursing and Midwifery, Faculty of Health, Deakin University, 1 Gheringhap Street, Geelong, Victoria, 3220, Australia; School of Nursing and Midwifery, Faculty of Health, Deakin University, 1 Gheringhap Street, Geelong, Victoria, 3220, Australia; School of Health and Social Development, Faculty of Health, Deakin University, 1 Gheringhap Street, Geelong, Victoria, 3220, Australia; The Institute for Mental and Physical Health and Clinical Translation, Deakin University, School of Medicine, 199 Ryrie Street Geelong, Victoria, 3220, Australia; School of Nursing and Midwifery, Faculty of Health, Deakin University, 1 Gheringhap Street, Geelong, Victoria, 3220, Australia; Centre for Quality and Patient Safety Research in the Institute for Health Transformation (IHT) – Western Health Partnership, Deakin University, Sunshine Hospital, 176 Furlong Road, St Albans, Victoria, 3021, Australia; Department of Public Health, Faculty of Health and Medical Sciences, University of Copenhagen, Blegdamsvej 3B, Copenhagen, 2200, Denmark; Faculty of Health Sciences, University of Southern Denmark and Steno Diabetes Center, Campusvej 55, Odense, 5230, Denmark; Department of Public Health, Aarhus University, Bartholins Alle 2, 2. sal, Aarhus, 8000, Denmark; Centre for Social and Early Emotional Development, School of Psychology, Faculty of Health, Deakin University, 1 Gheringhap Street, Geelong, Victoria, 3220, Australia; Centre for Adolescent Health, Murdoch Children’s Research Institute, Royal Children’s Hospital, 50 Flemington Road, Parkville, Victoria, 3052, Australia; Department of Paediatrics, Faculty of Medicine, Dentistry and Health Sciences, University of Melbourne, 50 Flemington Road, Parkville, Victoria, 3052, Australia

**Keywords:** health literacy, men, parent, health service settings

## Abstract

During pregnancy and early fatherhood, men are at higher risk of poor health, exacerbated by low engagement by healthcare services. Yet the transition to fatherhood presents an opportunity for men to improve their health and health behaviours. Health literacy refers to individuals’ competence in accessing and applying health information. Poor health literacy is associated with poor health and low help-seeking. The aim of this study was to identify health literacy strengths, needs and profiles among fathers. Men who were expecting a baby (‘antenatal’) or had become fathers in the past 18 months (‘postnatal’) were recruited through an international, online paid survey platform. The survey included the nine-scale Health Literacy Questionnaire (HLQ). Of 889 survey respondents (*n* = 416, 46.5% antenatal; *n* = 473, 53.5% postnatal), 274 (31.0%) were residing in the USA and 239 (27.0%) in the UK. Relatively higher scores were reported for HLQ scales relating to having sufficient information and finding and understanding this information, as well as social support for health. Relatively lower scores were obtained for scales relating to actively managing one’s own health and navigating the health care system. Three scale scores were significantly lower among nulliparous than multiparous men. Seven health literacy profiles were identified. In conclusion, while fathers have some health literacy strengths, they also experience some barriers, particularly first-time fathers. Awareness of diverse health literacy profiles among fathers may assist in developing strategies to strengthen health services’ capacity to meet fathers’ needs and reduce risks to their health at this critical juncture in families’ lives.

**Contribution to Health Promotion**

- Health literacy, one’s ability to find, use and apply health information, can improve health and health behaviours.
- Fathers’ health literacy strengths included finding and understanding health information.
- Fathers (especially first-time fathers) reported difficulties with finding their way around the healthcare system, managing their health and feeling supported by healthcare providers.
- Fathers’ needs should be considered in pregnancy and postnatal health care; this could improve fathers’ and families’ health at this important time.

## INTRODUCTION

Pregnancy and the birth of a baby can have profound effects on the physical (Garfield *et al*., 2006; Saxbe *et al*., 2018) and mental (Cameron *et al*., 2016; Leach *et al*., 2016) health of men, who are also often highly motivated at that time to improve their health (Baldwin *et al*., 2018; Rominov *et al*., 2018; Saxbe *et al*., 2018). New families have considerable contact with health services (Johnston *et al*., 2015). Thus, health professionals consulting with expectant and new parents have a unique opportunity to identify and support the partners of birthing mothers (Leahy-Warren *et al*., 2022), particularly those at risk of poor mental health. In this article, the focus is specifically on fathers (expectant and postnatal) given gendered considerations relevant to healthcare (The Lancet, 2019).

The experience of becoming a father can be an opportunity to increase men’s health promotion, health and health behaviours (Garfield *et al*., 2010; Macdonald *et al*., 2022). However, evidence suggests that fathers’ engagement with health services during the perinatal period is low (Fletcher, 2009; Wells, 2016; Schuppan *et al*., 2019; Leahy-Warren *et al*., 2022). In addition to providing the opportunity for health professionals to intervene early to improve fathers’ health, fathers’ engagement in health services can facilitate better support for mothers (Plantin *et al*., 2011), partner relationship quality and coparenting relationships (Bruno *et al*., 2020), breastfeeding outcomes for infants (deMontigny *et al*., 2018; Abbass-Dick *et al*., 2019), father–infant relationships and parenting outcomes (Plantin *et al*., 2011; Lee *et al*., 2018).

### Health literacy and its importance

One barrier to health service engagement among fathers may be poor health literacy, characterized by limited ability and/or resources to support engagement with health information and healthcare. Health literacy is defined as a person’s competence in accessing, understanding, appraising and applying health information in order to make sound health decisions (Stormacq *et al*., 2020). Health literacy includes not only functional health literacy skills (e.g. having the capacity to read a brochure about a health condition or fill out a questionnaire for a healthcare provider) but also communicative/interactive skills (e.g. ability to actively engage and participate with healthcare providers) and critical health literacy (ability to critically analyse and apply information) (Nutbeam, 2000). While Nutbeam’s scheme was theory driven, Osborne *et al*. (2013) grounded the psychometric development of the commonly used Health Literacy Questionnaire (HLQ) in people’s lived experience, suggesting that generation of outcome data across a range of real-world settings would substantially advance the health literacy field (Osborne *et al*., 2013). The nine scales of the HLQ incorporate Nutbeam’s functional, interactive and critical levels of health literacy (Nutbeam, 2000; Osborne *et al*., 2013). However, given the validity-driven approach to its development, the HLQ’s nine conceptually distinct scales of health literacy enable the investigation of the needs and capabilities of groups of individuals (Osborne *et al*., 2013).

Poor health literacy is a modifiable risk factor associated with reduced health help-seeking (Oliffe *et al*., 2020), and therefore reduced support and poorer physical and mental health (Levy and Janke, 2016). Being male is a moderate risk factor for poor health literacy (von Wagner *et al*., 2007). In addition, health literacy can mediate the relationship between education and health outcomes (Friis et al. 2016). Thus, assessing and addressing health literacy needs among communities can counter inequity in health and healthcare delivery (Batterham *et al*., 2016).

### Health literacy among mothers and fathers

There is an emerging body of research exploring health literacy among pregnant women (Meldgaard *et al*., 2022) and mothers (Morrison *et al*., 2019). The focus on mothers in these studies aligns with the fact that empirical research on health literacy has so far relied on studies among individuals who are already connected to a health care system (Levy and Janke, 2016). Fathers represent a population that is less engaged in health services (Wells, 2016; Schuppan *et al*., 2019); health literacy barriers may be one causal factor in this low engagement.

At other life stages, men’s knowledge and attitudes towards their health have been shown to be significantly, positively related to their intention to support their partners’ decision-making to make positive healthcare decisions (Dsouza *et al*., 2022). Not only is the perinatal period a critical window to improve men’s own health, but men can also play a critical role in health behaviour and decision-making around family health during this period (Martin *et al*., 2007; Judith and Jo, 2015; Aborigo *et al*., 2018; Lee *et al*., 2018). Meeting men’s health literacy needs at this time therefore has the potential to influence women’s engagement with healthcare during the critical transition to parenthood in addition to improving men’s own engagement in health services (Tokhi *et al*., 2018).

### Aims

The overall aim of this study was to investigate, for the first time and using a multidimensional approach, health literacy among fathers. Specifically, within a multi-country sample of fathers, the aim was to measure health literacy in expectant and new fathers, in order to (i) describe health literacy strengths and needs, including any differences according to parity and (ii) describe profiles of health literacy among groups of men, to demonstrate challenges faced by specific subpopulations of fathers. Such profiles can inform the development of effective strategies to improve health services’ ability to respond to these groups’ needs.

## METHODS

### Design

This was a multi-country, cross-sectional, observational study.

### Setting

Because fathers are not routinely engaged in health services, it can be challenging to recruit them to research studies (Leach *et al*., 2019). Participants were recruited via Prolific (www.prolific.co/), an online research recruitment platform based in the UK. It is open to participants from 34 countries across the world. Surveys in any language can be hosted on Prolific. Participants are paid for completing surveys based on time taken (at or above UK minimum wage); in this study, the median (range) of payment was £1.83 (£0.79–£8.52) for the pregnancy survey and £2.56 (£1.46–£8.42) for the postnatal survey. Data collection occurred between 22 August and 18 September 2021.

### Procedure

Prolific has over 130 000 potential participants who have been recruited via word of mouth, social media and flyers at universities (www.prolific.co/). Researchers enter specific eligibility criteria; for this study, these were men with pregnant partners or whose partners had had a baby since the beginning of 2020. Only potential participants with a Prolific account and those who met the study’s eligibility criteria were notified by email by Prolific and provided with the survey link, should they wish to participate. A link to the Plain Language Statement was included in the introduction to the survey; this could be downloaded by participants. Eligibility was assessed by screening questions at the beginning of the survey confirming that participants were over the age of 18, could read English and that partners were currently pregnant (pregnancy survey) or had had a baby since the beginning of 2020 (postnatal survey). Consent to participate was also confirmed with an additional question at the beginning of the survey.

### Participants

Men (from any country) with partners expecting a baby or whose partners had given birth since the beginning of 2020 were eligible to participate if they were over the age of 18 and could read English.

Of the 2 218 eligible account-holders at Prolific at the time, 1 003 commenced the survey. Data were discarded if respondents answered demographic questions but did not commence the HLQ. Of the 889 responses that remained, 413 (47%) were from men with a pregnant partner, while 476 (53%) had an infant born since the beginning of 2020.

Table 1 shows the demographic and reproductive characteristics of the study sample. Three-quarters of respondents were residing in the USA (31%), the UK (27%) or South Africa (18%); the rest reported residing in other countries in Europe, North and South America, Australasia and Asia. Although most respondents (85%) had completed some education after secondary school, a broad range of self-reported social standing [from 1 to 10, interquartile range (IQR) 5–7] was represented.

**Table 1:** Demographic, health and reproductive characteristics

Age in years (*n* = 888)	
Mean (SD)	32.5 (6.0)
Median (IQR)	32.0 (28.0–36.0)
Minimum, maximum	18.0, 57.0
Country of birth (*n* = 889)	*n* (%)
USA	259 (29.1)
UK	203 (22.8)
South Africa	139 (15.6)
Other	278 (32.5)
Country of residence (*n* = 885)	
USA	274 (31.0)
UK	239 (27.0)
South Africa	155 (17.5)
Other	219 (24.5)
Highest level of education completed (*n* = 889)	
Completed primary/elementary school	3 (0.3)
Completed secondary school	126 (14.2)
Completed trade school/apprenticeship/ diploma	125 (14.1)
Completed undergraduate degree	319 (35.9)
Completed postgraduate degree	316 (35.5)
Social standing^a^ (*n* = 885)	
Mean (SD)	5.9 (1.5)
Median (IQR)	6.0 (5.0 – 7.0)
Minimum, maximum	1.0, 10.0
Relationship status (*n* = 888)	
Not currently in a relationship	11 (1.2)
In a relationship, but not living together	103 (11.6)
Cohabiting: De facto	162 (18.2)
Cohabiting: Married	612 (68.9)
Self-reported physical health (*n* = 886)	
Good, very good or excellent	816 (91.9)
Poor or fair	72 (8.1)
Self-reported mental health (*n* = 886)	
Good, very good or excellent	738 (83.3)
Poor or fair	148 (16.7)
Partner pregnant or already given birth (*n* = 889)	
Pregnant	413 (46.5)
With first baby	137 (33.2)
Already given birth	476 (53.5)
First baby	239 (50.2)
*(If partner pregnant)* gestation in weeks (*n* = 413)	
<12	74 (17.9)
12–24	132 (32.0)
25–34	132 (32.0)
>34	75 (18.2)
*(If partner already given birth)* baby age in months (*n* = 444)	
Mean (SD)	10.6 (6.2)
Median (IQR)	11.0 (5.0 – 16.0)
Minimum, maximum	0.0, 36.0
Model of care during pregnancy (*n* = 877)	
Private (private obstetrician, privately practising midwife with planned home birth, private obstetrician and privately practising midwife joint care, privately practising midwife with planned hospital birth)	254 (29.0)
GP obstetrician care	47 (5.4)
Shared care GP and hospital	110 (12.5)
Public hospital (public hospital maternity care—mostly midwives, public hospital maternity care—mostly doctors)	411 (46.9)
Team midwifery or midwifery group practice (MGP) caseload care	44 (5.0)
Remote area maternity care	4 (0.5)
My partner did not attend pregnancy check-ups	7 (0.8)

^a^0 suggests the lowest level of self-reported social standing, and 10 the highest.

### Data sources

The survey was hosted on Qualtrics, an online survey platform (Qualtrics, 2021). The survey included:

*Socio-demographic and reproductive characteristics*: Respondents were asked about their age, language spoken at home, partner status, and country of birth and residence. Responses to questions about countries of birth and residence were collapsed to represent the three most common countries, and ‘Other’. Given that social disadvantage is a risk factor for poor health literacy (Beauchamp *et al*., 2015), a question was included regarding highest level of education attained. Socioeconomic position was also measured using a modified form of the Macarthur Scale of Subjective Social Status (Adler *et al*., 2000): Respondents are provided with a scale measuring from 0 to 10 and instructed ‘At the top of the scale (9-10) are the people who are the best off—those who have the most money, the most education, and the most respected jobs. At the bottom (0-1) are the people who are the worst off—those who have the least money, least education, the least respected jobs, or no job. The higher up you are on this scale, the closer you are to the people at the very top; the lower you are, the closer you are to the people at the very bottom. Please select a value that most represents you’ (Adler *et al*., 2000). This measure was selected as this was an international study where currencies or other indicators of socio-economic status may differ widely across contexts.

Men were also asked whether their partners were expecting or had already given birth, how many children they had and who provides/provided most of their partner’s pregnancy care. Responses to the latter were recoded into a binary variable: models with and without relational continuity. Relational continuity is described as ‘an ongoing therapeutic relationship between a patient and one or more provider’ [(Haggerty *et al*., 2003), p. 1220] and, in the case of this study, includes private obstetrician, GP obstetrician care, private obstetrician and privately practising midwife joint care, shared care between the hospital and a GP, privately practicing midwives (with planned home/hospital birth), team midwifery care and midwifery group practice (MGP care). All other models of care were classified as not having relational continuity. Relational continuity was selected as a variable to be explored in the context of fathers’ health literacy as in studies of childbearing women, relational continuity has been identified as a facilitator of women’s engagement in maternity care, particularly for families of ethnically diverse, vulnerable or socially disadvantaged backgrounds (Beake *et al*., 2013; Noseworthy *et al*., 2013; Dove and Muir-Cochrane, 2014; Ebert *et al*., 2014), or complex pregnancy or health issues (Watkins *et al*., 2022).

*General health:* Respondents were asked to rate their own physical and mental health using two 5-point Likert scale questions, with response options Poor, Fair, Good, Very good and Excellent. These items are based on an item asking participants to self-rate their general health in a validated measure (Ware *et al*., 1996).

*Health literacy*: Health literacy was assessed using the multidimensional HLQ (Osborne *et al*., 2013) which includes nine scales, each of which is made up of four to six items (total 44 items). The first five scales are measured on a Likert scale with a range of 1 to 4 (Strongly disagree, Disagree, Agree, Strongly agree): 1. Feeling understood and supported by health care providers; 2. Having sufficient information to manage my health; 3. Actively managing my health; 4. Social support for health; and 5. Appraisal of health information. The last four scales are measured on a 5-point Likert scale (Cannot do, Very difficult, Quite difficult, Quite easy, Very easy): 6. Navigating the health care system; 7. Ability to actively engage with health care providers; 8. Ability to find good health information; and 9. Understanding health information enough to know what to do. Internal consistency is reported to be good (Cronbach’s α between 0.8 and 0.9 for each scale) (Elsworth *et al*., 2016). Scales 2, 8 and 9 broadly match Nutbeam’s functional health literacy level; Scales 1, 3, 4, 6, 7 and 8 align with Nutbeam’s communicative/interactive health literacy level; and Scales 3, 4 and 5 fit within Nutbeam’s critical health literacy (Nutbeam, 2000; Osborne *et al*., 2013). On each scale, a higher score reflects relatively higher health literacy. No cut-off points have been validated for HLQ scales; scores are assessed relative to scores from the sample overall.

### Statistical methods

Sample size was based on detecting a difference in HLQ scale scores between two groups (partner is pregnant or has already given birth). With 90% confidence intervals and 80% power, to detect a difference of approximately 0.1 on a HLQ scale and based on an existing study of health literacy among men (Beauchamp *et al*., 2015) assuming a population variance of 0.59, the required sample size was 730.

Frequencies and percentages are reported for socio-demographic, health and reproductive characteristics which were collected as categorical variables.

For continuous variables, means, medians and interquartile range (IQR) are reported. For the HLQ, each scale is scored and reported independently using means and standard deviations (SD) (Osborne *et al*., 2013); as per the authors’ instructions, scale scores are calculated only if there are two or fewer missing item responses in that scale (Beauchamp *et al*., 2017).

As this is the first time the HLQ has been used among fathers, internal consistency of each HLQ scale is reported using Cronbach’s α. Mean HLQ scores are provided for each scale, for the sample overall and for nulliparous men (partners pregnant with the first baby), primiparous men (with one baby) and multiparous men (with two or more children). Significant differences between these three groups were assessed using Kruskal–Wallis tests as distributions were significantly non-normal.

To address the second aim of characterizing groups of men with different health literacy profiles, hierarchical cluster analysis was conducted using Ward’s method for linkage (Ward, 1963). This method was used as it allows one to generate a number of cluster solutions, which can then be examined to identify the ‘optimal’ solution, i.e. the number of clusters that best represent the sample (Osborne *et al*., 2021). In addition, as the HLQ is increasingly used in diverse samples internationally, the possibility exists that data from this study can be compared with other studies that use the same scales and analysis in the future. A range of solutions was investigated, from 3 to 16 clusters (Osborne *et al*., 2021). Selection of the most appropriate cluster solution was based on four criteria: First, a scree plot was generated and examined based on the agglomeration schedule, using the ‘elbow’ of the plot as a guide to indicating the optimal number of clusters. Second, the SD within each scale and cluster was examined, ensuring that these were minimized (Osborne *et al*., 2021). SDs of greater than 0.6 could indicate that there are still significant subgroups within a cluster. Third, a one-way analysis of variance (ANOVA) was conducted for each scale score and the means for each cluster on each scale were examined, to assess the degree to which the clusters were distinct. Non-significant F-values would indicate that variables were not contributing to the cluster separation. Finally, distinct patterns of HLQ scale scores were identified, as well as differences in socio-demographic variables between clusters.

The statistical software IBM SPSS Statistics (version 27) (IBM Corp, 2020) was used for analysis.

### Ethics

Ethics approval to conduct the study was obtained from Deakin University’s Human Ethics Advisory Group—Health (HEAG-H 107_2021).

## RESULTS

Table 2 presents Cronbach’s α and mean HLQ scores for each scale for the study sample. Cronbach’s α ranged from 0.8 to 0.9.

**Table 2:** Internal consistency and mean (SD) HLQ scale scores

	Cronbach’s α	Mean (SD)	95% Confidence interval Lower Bound, Upper Bound	Nutbeam’s schema^a^	Mean (SD): Nulliparous	Mean (SD): Primiparous	Mean (SD): Multiparous	*p*
Scale range 1–4								
1. Feeling understood and supported by health care providers	0.8	3.0 (0.6)	2.9, 3.0	Interactive	2.8 (0.6)^b^	2.9 (0.6)	3.0 (0.6)^c^	0.005
2. Having sufficient information to manage my health	0.8	3.1 (0.5)	3.1, 3.1	Functional	3.0 (0.4)	3.1 (0.4)	3.1 (0.5)	0.352
3. Actively managing my health	0.8	2.9 (0.6)	2.9, 3.0	Interactive, Critical	2.9 (0.5)	2.9 (0.6)	3.0 (0.6)	0.512
4. Social support for health	0.8	3.1 (0.5)	3.0, 3.1	Interactive, Critical	3.0 (0.7)	3.1 (0.5)	3.1 (0.5)	0.525
5. Appraisal of health information	0.8	3.0 (0.5)	2.9, 3.0	Critical	2.9 (0.5)^b^	3.0 (0.5)	3.0 (0.51)^c^	0.034
Scale range 1–5								
6. Ability to actively engage with health care providers	0.9	4.0 (0.6)	3.9, 4.0	Interactive	4.0 (0.6)	3.9 (0.6)	4.0 (0.6)	0.192
7. Navigating the health care system	0.8	3.9 (0.6)	3.8, 3.9	Interactive	3.8 (0.6)^b^	3.9 (0.6)	3.9 (0.6)^c^	0.033
8. Ability to find good health information	0.8	4.1 (0.5)	4.0, 4.1	Functional, Interactive	4.1 (0.5)	4.1 (0.5)	4.0 (0.6)	0.967
9. Understanding health information enough to know what to do	0.8	4.1 (0.5)	4.1, 4.2	Functional	4.1 (0.5)	4.1 (0.5)	4.2 (0.6)	0.236

^a^Functional, interactive or critical health literacy (Nutbeam, 2000).

^b^Groups that were significantly different in *post hoc* tests taking into account Bonferroni corrections

^c^Groups that were significantly different in *post-hoc* tests taking into account Bonferroni corrections.

### Aim (a): describe health literacy strengths and needs

For scales 1–5 with a highest possible mean score of 4, the highest scores were for Scale 2, ‘Having sufficient information to manage my health’ and Scale 4, 'Social support for my health' (3.1 ± 0.5), while the lowest score was for Scale 3, ‘Actively managing my health’ (2.9 ± 0.5). Relative to mean scores for other scales, lower mean scores were also observed for Scale 1, ‘Feeling understood and supported by health care providers’ (3.0 ± 0.6), and Scale 5, ‘Appraisal of health information (3.0 ± 0.5). For scales 6–9 with the highest possible mean score of 5, the highest scores were for Scale 8, 'Ability to find good health information' and Scale 9, ‘Understanding health information enough to know what to do’ (4.1 ± 0.5) and the lowest score was for Scale 7, ‘Navigating the health care system’ (3.9 ± 0.6).

On 3 scales, nulliparous men had significantly lower scores than multiparous men (Table 2).

### Aim (b): describe profiles of health literacy among fathers

Hierarchical cluster analysis revealed seven distinct health literacy profiles (Table 3). ANOVA showed significant differences between clusters for each HLQ scale score (*p* < 0.001).

**Table 3:** Health literacy, socio-demographic and health characteristics by cluster (*n* = 883)

Cluster	A	B	C	D	E	F	G	All
*n* in cluster (% of sample)	115 (13.0)	132 (15.0)	243 (27.5)	108 (12.2)	104 (11.8)	96 (10.9)	85 (9.6)	883 (100.0)
1. Healthcare provider support	3.7 (0.3)	3.1 (0.5)	3.1 (0.4)	2.7 (0.4)	2.6 (0.5)	2.7 (0.4)	2.7 (0.4)	3.0 (0.6)
2. Having sufficient information	3.7 (0.3)	3.3 (0.4)	3.0 (0.3)	2.9 (0.2)	3.0 (0.3)	2.7 (0.4)	3.0 (0.2)	3.1 (0.5)
3. Actively managing health	3.5 (0.4)	3.1 (0.4)	3.1 (0.4)	2.4 (0.4)	3.0 (0.3)	2.3 (0.5)	3.1 (0.4)	2.9 (0.6)
4. Social support	3.7 (0.3)	3.2 (0.3)	3.2 (0.3)	3.0 (0.3)	2.7 (0.5)	2.7 (0.4)	2.6 (0.4)	3.1 (0.5)
5. Critical appraisal	3.6 (0.3)	3.0 (0.4)	3.1 (0.3)	2.6 (0.4)	2.9 (0.3)	2.6 (0.5)	2.8 (0.5)	3.0 (0.5)
6. Active engagement with healthcare providers	4.7 (0.3)	4.4 (0.4)	4.0 (0.3)	4.1 (0.3)	3.5 (0.4)	3.6 (0.4)	3.1 (0. 5)	4.0 (0.6)
7. Navigating the healthcare system	4.6 (0.3)	4.3 (0.4)	3.8 (0.4)	4.0 (0.3)	3.6 (0.4)	3.5 (0.3)	3.2 (0.4)	3.9 (0.6)
8. Ability to find good health information	4.7 (0.3)	4.4 (0.4)	4.0 (0.3)	4.2 (0.4)	4.0 (0.4)	3.6 (0.4)	3.1 (0.4)	4.1 (0.5)
9. Reading and understanding health information	4.7 (0.3)	4.6 (0.3)	4.0 (0.3)	4.3 (0.4)	4.1 (0.3)	3.8 (0.4)	3.1 (0.4)	4.1 (0.5)
Age (years), mean (SD)	34.0 (5.8)	33.6 (6.1)	32.0 (6.6)	32.5 (4.5)	31.8 (5.6)	32.3 (6.0)	31.4 (5.6)	32.5 (6.0)
Speak English at home, *n* (%)	107 (93.9)	114 (86.4)	203 (83.9)	95 (88.0)	87 (83.7)	81 (84.4)	70 (82.4)	757 (85.9)
Completed university degree, *n* (%)	97 (84.3)	107 (81.1)	179 (73.7)	63 (58.3)	75 (72.1)	60 (62.5)	52 (61.2)	633 (71.7)
Self-reported social standing, mean (SD)	6.6 (1.4)	6.1 (1.3)	6.1 (1.4)	5.7 (1.4)	5.8 (1.5)	5.5 (1.4)	5.3 (1.5)	5.9 (1.5)
Cohabiting with a partner, *n* (%)	100 (87.7)	115 (87.1)	217 (89.3)	97 (89.8)	84 (80.8)	84 (87.5)	74 (87.1)	771 (87.4)
Country of residence								
USA, *n* (%)	69 (60.5)	37 (28.2)	91 (37.6)	15 (13.9)	22 (21.2)	19 (20.0)	21 (24.7)	274 (31.2)
UK, *n* (%)	11 (9.7)	37 (28.2)	46 (19.0)	55 (50.9)	39 (37.5)	29 (30.5)	21 (24.7)	238 (27.1)
Postpartum, *n* (%)	44 (38.3)	70 (53.0)	121 (49.8)	71 (65.7)	63 (60.6)	60 (62.5)	45 (52.9)	474 (53.7)
Number of children								
0	13 (11.3)	17 (12.9)	39 (16.0)	19 (17.6)	17 (16.3)	14 (14.6)	13 (15.3)	132 (14.9)
1	43 (37.4)	53 (40.2)	104 (42.8)	49 (45.4)	50 (48.1)	38 (39.6)	40 (47.1)	377 (42.7)
2 or more	59 (51.3)	62 (47.0)	100 (41.2)	40 (37.0)	37 (35.6)	44 (45.8)	32 (37.6)	374 (42.4)
Model of care: Models with relational continuity^a^, *n* (%)	70 (60.9)	72 (55.0)	142 (58.4)	56 (51.9)	46 (46.0)	40 (42.6)	29 (35.4)	455 (52.1)
Physical health: Good, very good or excellent, *n* (%)	113 (98.3)	126 (95.5)	225 (93.0)	101 (93.5)	96 (92.3)	78 (81.3)	71 (83.5)	810 (91.8)
Mental health: Good, very good or excellent, *n* (%)	109 (95.6)	109 (83.2)	214 (88.4)	94 (87.0)	74 (72.1)	71 (74.0)	60 (70.6)	732 (83.2)

^a^An ongoing relationship between a patient and one or more care providers (Haggerty *et al*., 2003).

The clusters indicated some distinct profiles among participants, each with specific strengths and barriers:

Cluster A (13.0% of the sample) **Active, able, and supported.** This group scored the highest means on all HLQ scales and reported good physical and mental health (Table 3). A relatively low proportion of men in this group were postpartum (38.3%); a relatively high proportion reported speaking English at home (93.9%) and had a university degree (84.3%). The mean self-reported social standing (6.6) was higher than in other groups. In this group, a high proportion lived in the USA (60.5%) and a low proportion in the UK (9.7%). The proportion of nulliparous men was lowest (11.3%), while the proportion of multiparous men was highest (51.3%). This cluster represented the group reporting the highest proportion of models of care with relational continuity (60.9%), good to excellent physical health (98.3%) and good to excellent mental health (95.6%).

Cluster B (14.9% of the sample) **Active and supported, some difficulty with critical literacy skills.** This cluster was similar to Cluster A with a high level of capacity, engagement and support across all scales. However, mean scores on Scales 3–5 were slightly lower than Cluster A, indicating slightly poorer critical literacy skills (Table 3).

Cluster C (27.5% of sample) **Mostly active and supported, some difficulty understanding health information.** This group scored moderately on most scales but compared to Clusters A and B, reported reduced capacity to understand written health information (Scale 9). This group reported a relatively high mean self-reported social standing (6.1).

Cluster D (12.2% of sample) **Confident with health information, but not proactive or critical.** This cluster scored moderately on most scales. They were confident users of the health system and health information (Scales 6–9) but, compared to almost all other clusters, were less proactive managers of their own health (Scale 3) and reported more difficulty with applying or critically appraising healthcare information (Scale 5) (Table 3). Compared to most groups, this group included a higher proportion of nulliparous men (17.6%), a higher proportion of men living in the UK (50.9%), a lower proportion living in the USA (13.9%), and a lower proportion with a university degree (58.3%).

Cluster E (11.8% of sample) **Less supported, less engaged, and less able to advocate for themselves.** This group scored moderately on most scales but compared to most other clusters, indicated feeling unsupported (Scales 1 and 4) as well as having limited ability to advocate for themselves or to look beyond obvious resources (Scales 6 and 7). Lower scores than most other clusters on Scales 1 and 6 indicated little trust or confidence to build relationships with health care providers (Table 3). The proportion of men who were cohabiting with a partner was relatively low (80.1%). Compared to other groups, this group had the highest proportion of first-time fathers (48.1%) and the lowest proportion of fathers with 2 or more children (35.6%). A relatively small proportion resided in the USA (21.2%). A relatively low proportion of men in this group reported good mental health (72.1%).

Cluster F (10.9% of sample) **Experiencing barriers to engaging in their own care**. This group scored relatively low on most scales, but were particularly likely to have gaps in knowledge, to be passive and disengaged when managing their own healthcare, to feel unsupported socially, and to be more easily confused by conflicting information (Scales 2–5) (Table 3). This group reported a relatively low mean self-reported social standing (5.5). Compared to most other groups, men in this group were also less likely to reside in the USA (20.0%). A relatively low proportion of men in this group reported having a university degree (62.5%) and good physical (81.3%) and mental (74.0%) health.

Cluster G (9.6% of sample) **Many health literacy barriers but somewhat active in managing their own healthcare**. This group scored lowest on some functional health literacy scales reflecting the ability to navigate the healthcare system, and to find, read and understand health information (Scales 7–9) as well as active engagement with healthcare providers (Scale 6). Compared to other clusters, they scored moderately on having sufficient information, actively engaging in their own healthcare, social support and critical appraisal (Scales 2–5) (Table 3). Of all the clusters, this group reported the lowest mean self-reported social standing (5.3). In this group, a relatively low proportion reported a university degree (61.2%), good physical health (83.5%) and good mental health (70.6%).

## DISCUSSION

This study aimed to describe the health literacy of men who were expecting a baby or who had become fathers in the past 18 months. This is the first study to investigate health literacy among a multi-national sample of fathers using a multidimensional measure that provides valuable insight into the health literacy strengths and needs of fathers.

### Aim (a): health literacy strengths and needs among fathers

Overall strengths were evident in fathers’ functional health literacy (Nutbeam, 2000); in most clusters, fathers reported having sufficient information to manage their health and understanding health information enough to know what to do. However, there remains considerable opportunity for health services to better meet fathers’ interactive and critical health literacy needs (Nutbeam, 2000). The relatively low scores, particularly among nulliparous men, regarding feeling understood and supported by healthcare providers, are consistent with men’s reports of not feeling included or welcome at antenatal and maternal health services (Jeffery *et al*., 2015; Wells, 2016). The lower scores regarding critical appraisal of health information align with fathers’ reports that they struggle to identify which support resources are good quality (Rowe *et al*., 2013; Entsieh and Hallström, 2016; Baldwin *et al*., 2018; Rominov *et al*., 2018; Schuppan *et al*., 2019). The skills required for critical analysis and application of health information may be harder to acquire than other health literacy skills; relatively low scores on this scale of the HLQ are not unusual (Beauchamp *et al*., 2015).

The relatively lower scores, particularly among nulliparous men, regarding men actively managing their own health and navigating the health system during the perinatal period are of concern as poor paternal mental health has negative consequences not only for fathers but also for their partners and infants (Paulson and Bazemore, 2010; Johnston *et al*., 2015; Sweeney and MacBeth, 2016). In many regions of the world, fathers accompany their partners and infants to health service consultations (Kotelchuck *et al*., 2022); an opportunity exists for government policymakers and healthcare leaders to identify and address fathers’ specific needs, particularly the needs of men whose partners are expecting their first baby. During the transition to fatherhood, fathers may be motivated to improve their own health for the sake of the baby (Garfield *et al*., 2010; Rominov *et al*., 2018). Understanding the health literacy abilities and needs of fathers will help health services to better engage this group at a critical time and improve outcomes for fathers, mothers and children.

### Aim (b): describe profiles of health literacy among groups of fathers

Health literacy profiles identified in this study ranged from men with higher health literacy who were confident users of health information and services (Cluster A), through to men with lower health literacy who require ongoing support to find, understand and use information to manage their health (Cluster G). In Cluster A, the cluster scoring highest mean scores on all scales, a relatively high proportion lived in the USA (61% compared to 31% of the overall sample) and reported models of care with relational continuity (61% compared to 52%). Importantly, no country of residence was prominent in Cluster G, the cluster with the lowest scores across all subscales, but only 35% of fathers in this cluster reported relational models of care. Given that relational models of care are often characterized by personal trust and responsibility (Jenkins *et al*., 2015), several researchers have reported benefits among mothers associated with relational continuity during the perinatal period, including: ‘feeling more than numbers’ (Jepsen *et al*., 2017), satisfaction with care (Haines *et al*., 2015; Forster *et al*., 2016), and improved perinatal mental health (Hildingsson *et al*., 2019). Further research is required into the impact of relational continuity upon health literacy and fathers’ experiences of care.

A greater understanding of fathers’ health literacy may assist in developing strategies to improve health services’ capacity to meet fathers’ diverse needs at this critical juncture in their own and their families’ lives. For example, among groups of men who are confident users of the health system and health information but report poor health literacy when it comes to applying or critically appraising healthcare information (e.g. Cluster D in this study), fathers’ health literacy strengths (*i.e.* their existing relationships with the health system) could be used to address some of the health literacy barriers they are experiencing, by providing guidance on where to find evidence-based sources of information and support, and how to translate these into behaviours.

Poor health literacy can be risk factor for poor health (Wolf *et al*., 2010; Pelikan *et al*., 2012; Levy and Janke, 2016; Aaby *et al*. 2017). Indeed, poorer physical and mental health was reported by fathers in the clusters with the lowest health literacy scores (Clusters D, E and F). For these groups of men, healthcare providers may need to initiate engagement and support, enabling them to feel more empowered and potentially preventing poor health outcomes among fathers, their partners and infants. Changes to clinical practice may require a cultural shift and buy-in from government and health service leaders to facilitate relationship-building between health professionals and fathers, who may not traditionally be considered part of the health service’s scope of practice. Not meeting the needs of fathers can have dire consequences for mothers’ mental health (Paulson and Bazemore, 2010) and children’s subsequent social, emotional and behavioural development (Sweeney and MacBeth, 2016).

The pattern of self-reported social standing rankings closely followed the health literacy profiles; clusters with higher (lower) health literacy scale scores reported higher (lower) self-reported social standing. Social disadvantage may be a risk factor for poor health literacy (Beauchamp *et al*., 2015); however, there is also evidence that poor health literacy may partially mediate the pathway from social disadvantage to poor health outcomes (Friis *et al*., 2016). Therefore, addressing fathers’ health literacy needs may be a pathway for improving health outcomes in disadvantaged groups.

### Strengths and limitations

This study addresses a gap in the existing evidence about parents’ health literacy. To date, no studies have investigated functional, interactive and critical health literacy among fathers. Internal consistency was adequate for all subscales of the HLQ, indicating that psychometric investigation of the HLQ as an appropriate measure to assess health literacy among fathers is warranted.

Recruiting through an international online platform allowed for adequate recruitment in a relatively short time frame. As participants were paid for their time, no difficulties were experienced recruiting fathers as is often reported in other studies (Leach *et al*., 2019). Importantly, sample diversity was achieved in terms of self-reported social standing.

Some limitations are acknowledged. First, the international nature of the sample means specific recommendations cannot be made to healthcare providers in any region of the world; rather the patterns indicated in the data demonstrate that in all communities, health literacy profiles and needs among fathers are likely to be complex. Second, HLQ data from community studies of men of reproductive age who were not fathers could not be located, with which to compare the data from this study. Third, the sample represents fathers who had registered with an online survey company; findings cannot be generalized to other fathers, for example those who do not have the time to participate in such surveys. The fact that participants are paid for participation and according to the duration of the survey is also an important limitation; participants who complete surveys for monetary reward would not necessarily be representative of expectant or new fathers more generally. Finally, this was a cross-sectional study; longitudinal studies are needed to track fathers’ health literacy across pregnancy and the postnatal period, and identify ‘touch points’ at which meeting fathers’ needs could improve health literacy and thus facilitate better health outcomes among fathers and their families.

## Conclusions

This study provides important baseline and reference data for other studies regarding functional, interactive and critical health literacy among fathers. It provides insights into some of the barriers that may reduce fathers’ access to, engagement with, and benefits from health information and health services during the antenatal and postnatal periods.

Differences across countries indicate that local data in specific country or model-of-care contexts may be informative. Based on data in response to a multidimensional tool such as the HLQ, it may be possible to identify groups of men with specific health literacy strengths and needs, and co-design strategies to meet the needs of men in the clusters. However, in clinical practice comprehensive screening may not be feasible. To limit the resources required in settings where individual assessments and tailored responses at an individual level may not be feasible, a ‘toolkit’ of avenues for resources and support for fathers could be developed and adapted as appropriate.

It is important that health professionals, health service leaders and government policy makers invested in perinatal health services are aware of the broad range of skills that contribute to health ‘literacy’. Health literacy is much broader and more nuanced than the ability to acquire medical knowledge (Oliffe *et al*., 2020). Healthcare leaders should consider funding models which facilitate health services’ responsiveness to the health literacy needs of distinct groups of men. Although health literacy as a program design consideration (Nutbeam et al., 2018) has shown promise in improving health outcomes in other contexts, such as cardiac rehabilitation (Beauchamp *et al*., 2020), the role of health literacy in the design of health services, programs and support is absent from the current discourse on engaging fathers in health services. Consideration of fathers’ health literacy strengths and needs may lead to improvement in their engagement by health services, with benefits for their physical and mental health and that of their partners and infants.
